# Correction

**DOI:** 10.1111/cas.15012

**Published:** 2021-07-02

In an article^1^ titled “Blockade of ONECUT2 expression in ovarian cancer inhibited tumor cell proliferation, migration, invasion and angiogenesis” by Tongyi Lu, Binhua Wu, Yunfei Yu, Wenhui Zhu, Simin Zhang, Yinmei Zhang, Jiaying Guo, and Ning Deng, the following errors were published:

- In Figure 3C (c), the scratch image of siRNA#2‐24h in the scratch test has been replaced. The correct figure is presented below.

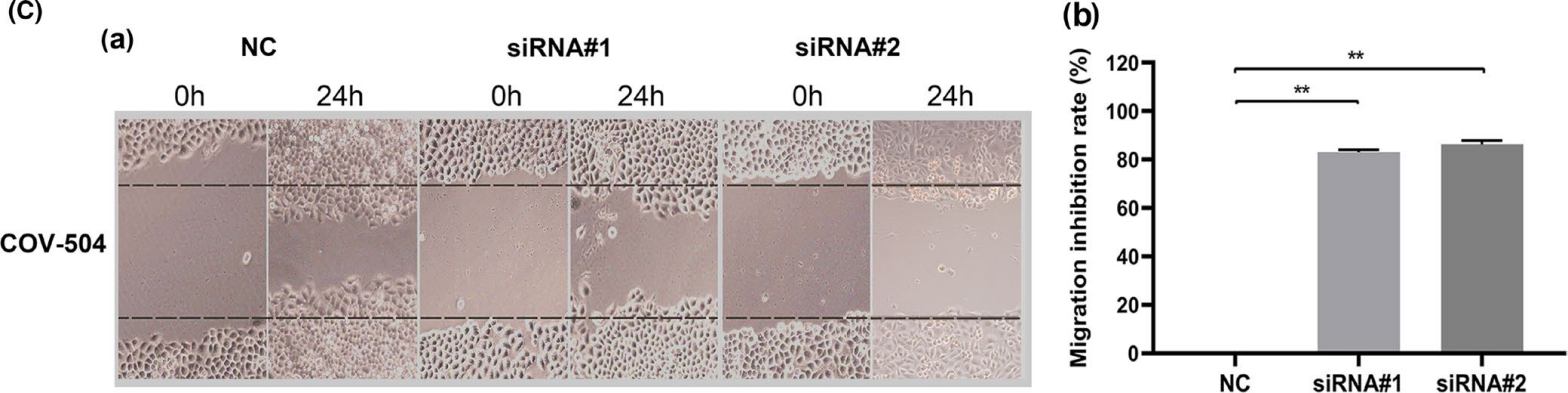

- In Figure 5A, the ACTB strips have been replaced. The correct figure is presented below.

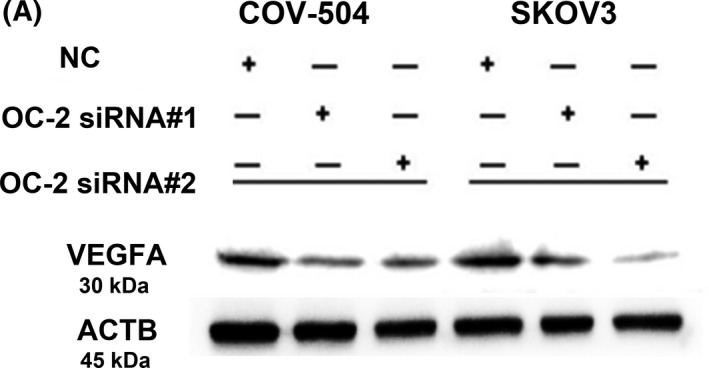

- In Figure 6A (a), the ACTB and ERK strips have been replaced. The correct figure is presented below.

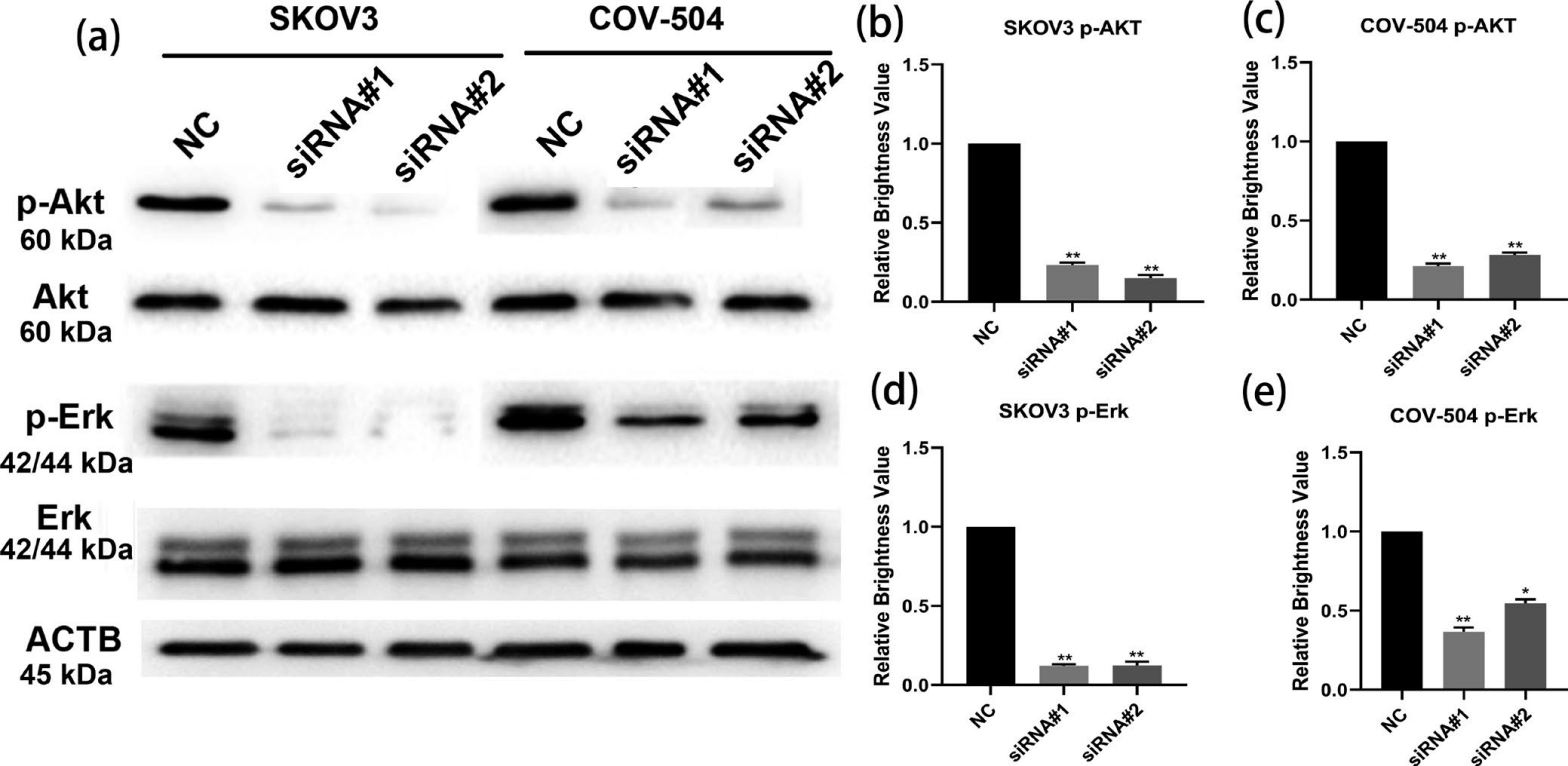

The authors apologize for the errors.
